# Prototype posterior chamber phakic IOL – 35 year follow up^☆^

**DOI:** 10.1016/j.ajoc.2024.102086

**Published:** 2024-06-01

**Authors:** Spyridon Doumazos, Aikaterini Barlampa, Stylianos A. Kandarakis, Menelaos Kanakis, George Kymionis, Ilias Georgalas, Petros Petrou

**Affiliations:** a First Department of Ophthalmology, National and Kapodistrian University of Athens, “G. Gennimatas” General Hospital of Athens, Greece; b Department of Ophthalmology, University of Patras, University Hospital of Patras, Greece

**Keywords:** Posterior chamber-Phakic IOL, Cataract complications, Prototype phakic IOL, Fyodorov, Lensectomy, Vitrectomy

## Abstract

**Purpose:**

To present a case involving a rarely seen prototype posterior chamber phakic IOL (PC-pIOL) in a highly myopic patient with bilateral cataract.

**Observations:**

A 64-year-old male presented to our clinic with poor vision in both eyes. Clinical examination revealed bilateral mature cataract, phacodonesis as well as a PC-pIOL implanted 35 years ago to address his high myopia. The visual acuity (VA) was 20/200 in the right eye and no light perception in the left eye. PC-pIOL extraction as well as 23G pars plana vitrectomy (PPV) and fragmentation surgery was scheduled for the right eye. The left eye was treated conservatively. Successful extraction of the PC-pIOL was performed while it was easy to remove. It was a bow-tie shaped lens with a collar-stud-like button in the middle which extended anteriorly into the anterior chamber through the pupil. PPV with lens fragmentation was successful and the patient was left aphakic in order to avoid the placement of a zero diopter IOL. Final best corrected VA was 20/25 one month post-surgery.

**Conclusions and importance:**

Removal of this rarely seen pIOL was performed without difficulty while excellent VA was achieved. Aphakia following complete vitrectomy represented a viable option in this case. Furthermore, we highlight the clinical manifestations associated with this IOL more than three decades after implantation.

## 1 Introduction

Phakic intraocular lenses (pIOL) are intraocular devices which are implanted in order to correct high levels of ametropia and anisometropia while preserving the crystalline lens.^1^ There are two main categories of pIOLs, anterior (AC) and posterior chamber (PC) pIOLs. AC pIOLs are either iris or angle fixated.^2^^,^^3^

PC-pIOLs were designed to be placed between the iris and the anterior surface of the crystalline lens.^1^ In 1986 Svyatoslav Fyodorov et al. were the first to design and implant such a lens in the former Soviet Union.^4^ Specifically, they designed a prototype which was not purely a PC-pIOL but rather an AC-PC pIOL or pupil-fixated lens.^4^^,^^5^ It was a one-piece silicone (teflon-coated) lens that had a bow-tie shape with a 3.5mm collar-stud-like button (optic) in the middle which extended into the AC through the pupil when in situ (Fig. 1A, Fig. 1BA–B).^1^^,^^4^

**Fig. 1A:**
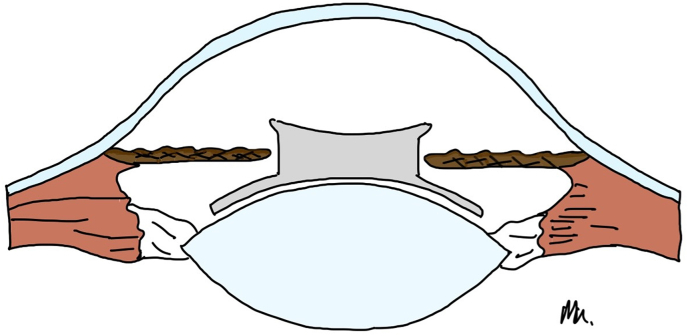
Illustration of the prototype pIOL in situ (Side view-not to scale). Optic is in position within the anterior chamber while the haptics are positioned in the posterior chamber.

**Fig. 1B:**
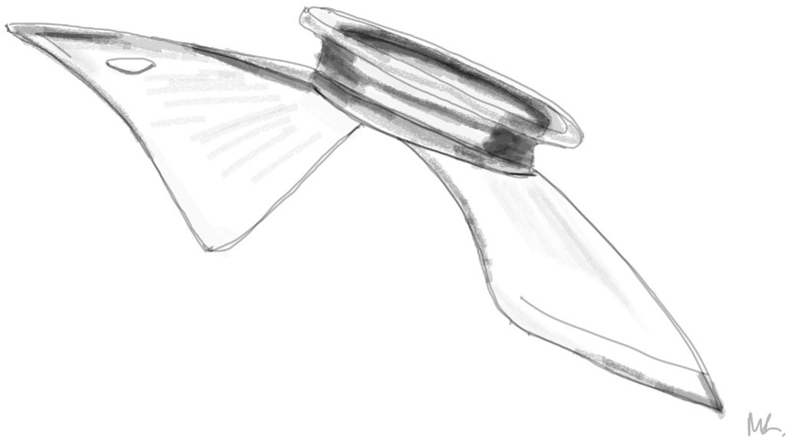
Illustration of the prototype pIOL. (Side view).

We herein present a more than three decades follow up of such case with it's clinical manifestations and our surgical approach to the patient's management.

## 2 Case report

A 64-year-old male presented to our clinic complaining of progressively deteriorating vision bilaterally while he neglected to seek ophthalmic care for the past few years. Clinical examination revealed a bilateral mature cataract as well as a PC-pIOL implanted in both eyes. According to the patient this pIOL was implanted 35 years ago in order correct his high degree myopia (−19.0sph). He had a history of glaucoma and was under maximum medical treatment (Latanoprost, Dorzolamide-Timolol and Brimonidine) in both eyes. As reported by the patient the glaucoma treatment started many years after his refractive surgery.

The corrected distance VA (CDVA) of the right eye was 20/200 on the Snellen chart, while the slit lamp examination revealed a deep anterior chamber, a nuclear sclerotic and anterior sub-capsular mature cataract, phacodonesis, a patent peripheral iridotomy and an IOP measurement of 16 mmHg. There was no perception of light (NPL) of the left eye and the slit lamp examination demonstrated a deep anterior chamber, neovascularization of the iris, a patent peripheral iridotomy, nuclear sclerotic mature cataract, phacodonesis and an IOP of 36 mmHg without pain (Fig. 1C, Fig. 1D, Fig. 1E, Fig. 1FC–F). B-scan examination of both eyes was unremarkable. The axial length of the right eye was 30.32mm, the anterior chamber depth was 3.09mm and the endothelial cell density (ECD) was 1556 cells/mm^2^.

**Fig. 1C:**
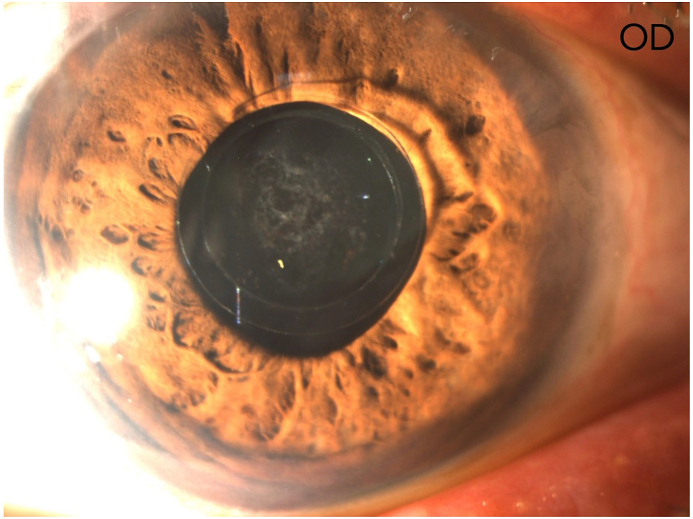
Preoperative picture of the right eye which is in mid-dilation showing the haptic-optic junction as well as the anterior sub capsular opacities.

**Fig. 1D:**
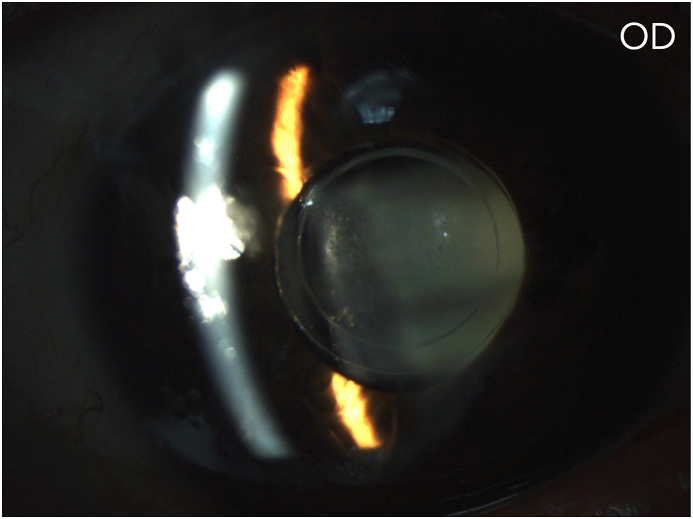
Preoperative picture of the right eye in mid-dilation shown under slit-lamp examination indicating a deep anterior chamber, an anterior subcapsular and nuclear cataract.

**Fig. 1E:**
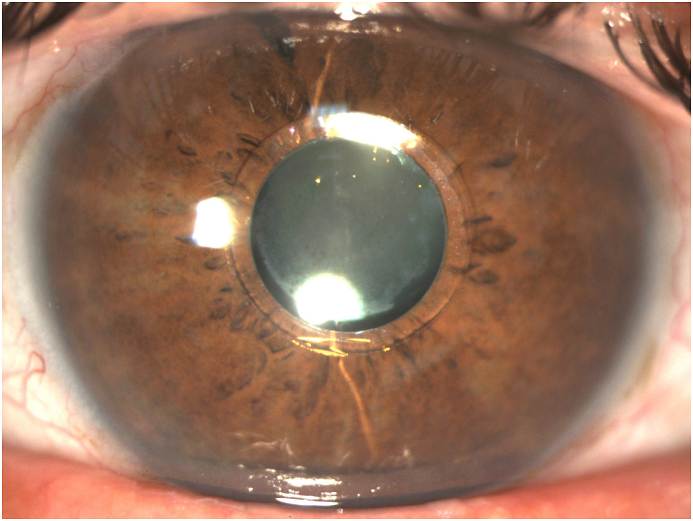
Preoperative picture of the right eye showing the central optic fixated at the pupil as well as the patent iridotomy at the upper quadrant.

**Fig. 1F:**
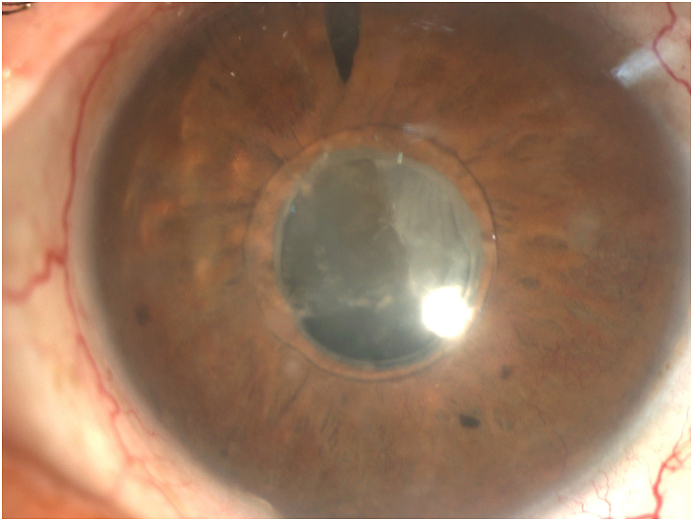
Preoperative picture of the left eye showing the central optic fixated at the pupil, the patent iridotomy as well as neovascularization of the iris.

After obtaining informed consent, extraction of the PC-pIOL as well as pars plana vitrectomy and lens fragmentation was scheduled for the right eye, see attached video (Video 1) (Fig. 1H). AS-OCT was performed as part of the pre-operative examination to evaluate the size and shape of this pIOL since it has been discontinued for over 30 years (Fig. 1G). The patient was left aphakic since a scleral fixated IOL of zero diopters was needed for emmetropia. The immediate post operative IOP was normal (13 mmHg) while there was no IOP spike. Fundoscopic findings were compatible with high myopia as well as a cup-disc (C/D) ratio of 0.6.

**Fig. 1G:**
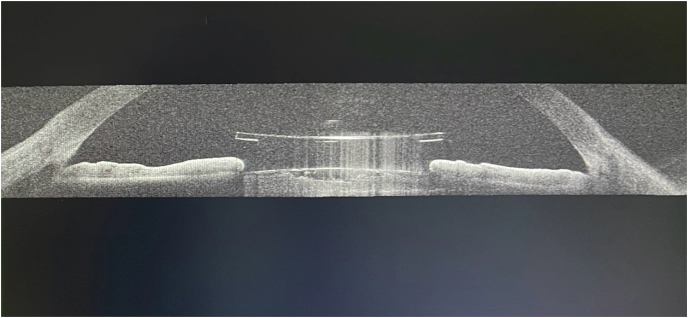
Preoperative AS-OCT of the right eye showing the central optic protruding through the pupil into the anterior chamber.

**Fig. 1H:**
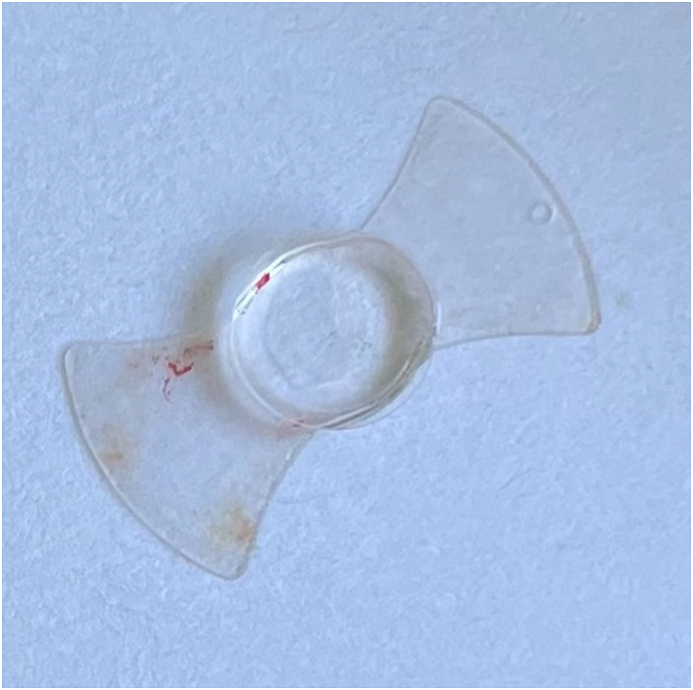
Prototype “Bow-tie” Fyodorov PC-pIOL removed from our patient.

The six months post-operative follow-up showed a CDVA of 20/25 with clear cornea (Fig. 1I). The rest of the examination was unremarkable. The anti-glaucoma drops eventually reached the same maximum regiment post operatively to be able to maintain an IOP of 12 mmHg. The postoperative visual fields of the right eye are shown in Fig. 2.

**Fig. 1I:**
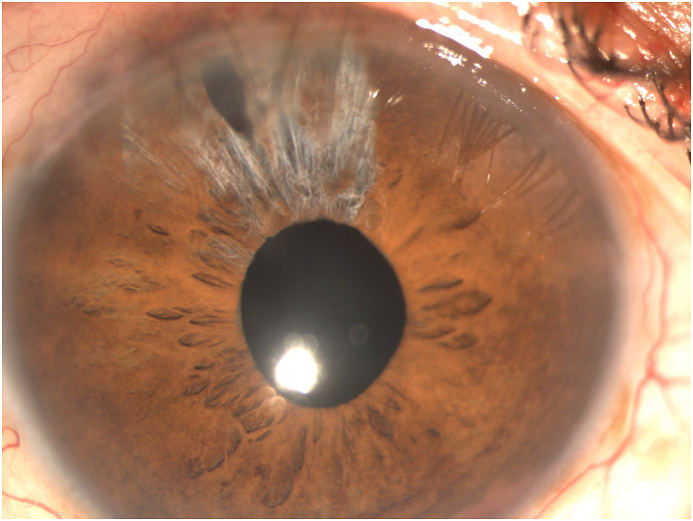
Postoperative picture of the right eye after removal of the pIOL showing aphakia.

**Fig. 2:**
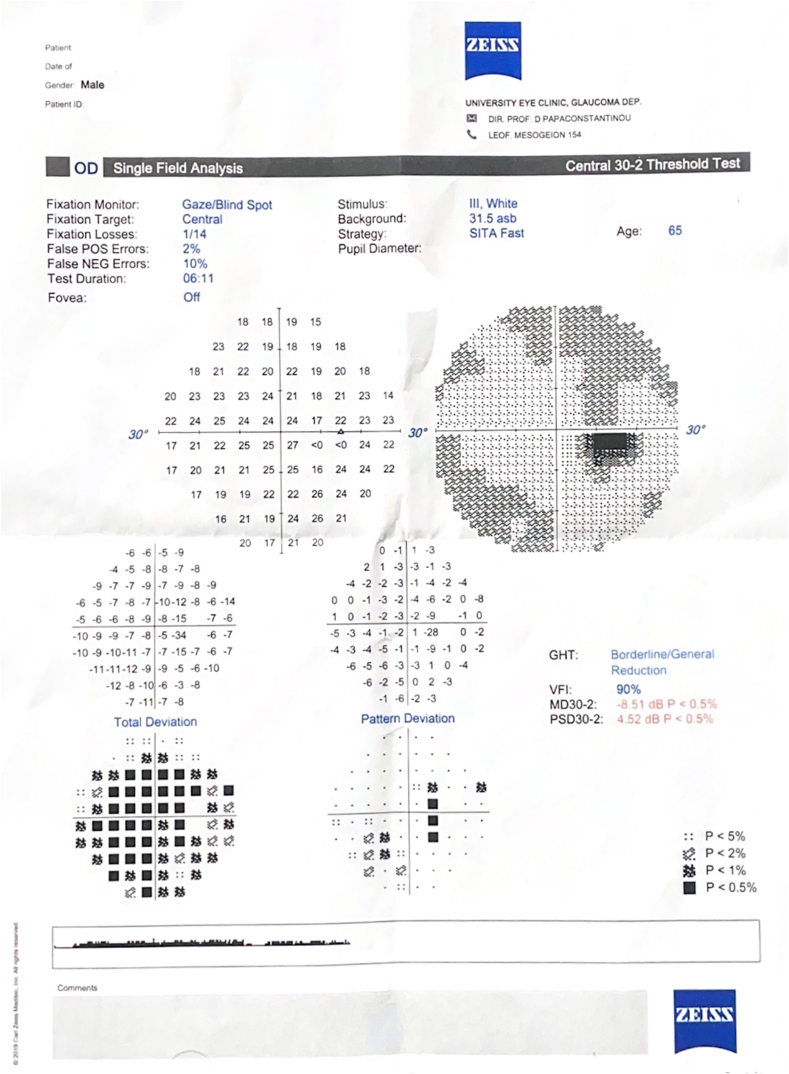
Visual fields of the right eye after surgery.

After conducting a literature review on (December 20, 2023) utilizing PubMed and Google Scholar using the key words (“Fyodorov phakic IOL”, “First generation posterior chamber-Phakic IOL″, “Top-Hat style phakic IOL” “Sulcus fixated IOL","Phakic IOL Complications",“Phakic IOL follow up”), we did not find any prior reports with more than 35 years of follow-up after this specific prototype fyodorov PC-Phakic IOL insertion. We believe this makes our report interesting and accentuates its importance.

## 3 Discussion

The need to surgically treat high myopia in young patients led to the development of this AC-PC pIOL by Fyodorov et al. which was used between 1986 and 1990.^4^^,^^5^ This lens enabled effective and efficient correction of high myopia while not affecting the accommodating ability of the natural lens and being in the PC had potentially fewer corneal side effects.^5^ As with our patient, fast and long-lasting visual recovery was achieved.

However, various complications led to its discontinuation. Pupillary block glaucoma, uveitis, corneal decompensation and mainly cataract formation were among the complications reported.^1^^,^^4^^,^^5^ Moreover, due to the small diameter of the optic (3.2mm) situated into the AC, nighttime visual disturbances have been reported as well as photophobia during bright light since the pupil was unable to constrict further than the optic diameter.^5^ In a meta-analysis published by Chen et al., in 2008 the incidence of cataract formation after insertion of various PC-pIOL models was 9.6 % while the most common reason for pIOL removal was cataractogenesis.^2^ Brauweiler P et al. reported an astonishing 82 % incidence of cataract formation in patients with implantation of the so-called Adatomed fyodorov pIOL which was one generation after our case's prototype, while the author discouraged future insertion of such lens.^6^ Conversely, Kohnen et al. in a 10-year follow-up of a patient after bilateral insertion of the Adatomed fyodorov lens reported no cataract formation and supported that the space between the natural lens and the pIOL is vital to avoid cataractogenesis, thus good IOL positioning.^7^

Regarding the prototype lens as in our case, Wiechens B et al. reported a case of bilateral cataract formation within 7 years after implantation of the pIOL.^8^ On the other hand, Bozkurt E et al. reported an 18-year follow up after the insertion of the same IOL in a patient without cataract formation but rather a more than 50 % ECD loss since insertion of the pIOL.^9^

Interestingly, although numerous reports suggest that cataractogenesis is a major short-term complication of PC-pIOLs our patient, similarly to Bozkurt E et al., had no complains with his VA and only recently he noticed deterioration of his vision.^3^^,^^5^^,^^9^ This means that according to our patient he spent more than 30 years with good VA. Thus, the cataract formation may not be solely attributed to the pIOL as suggested by the literature, but possibly due to a combination of factors. Notably, the implantation method seems to be vital since iatrogenic cataractogenesis can be initiated as well as the improper placement or size of the IOL.^5^^,^^7^ Regarding the ECD, no major cell loss was witnessed in the patient's right eye even after our intervention (1419 cells/mm^3^) while on the left eye the ECD was critically low albeit in an eye with NLP (790 cells/mm^3^).

Glaucoma is another complication that is linked with the insertion of phakic IOLs.^3^^,^^5^ Our patient developed neovascular glaucoma (NVG), however, we cannot determine whether the pIOL played a role in the pathogenesis. High myopia is a known risk factor for primary open angle glaucoma (POAG) development independent from intraocular interventions.^5^ POAG increases the risk of neovascularization in the presence of ocular ischaemia. Potentially more frequent monitoring of the patient's condition could have prevented the development of NVG. Due to the design of the pIOL multiple mechanisms could have been involved in the development of glaucoma, including UGH syndrome, pigment dispersion as well as pupillary block since the timing of iridotomies could not have been specified. However, there were no transillumination defects of the iris pertaining to pigment dispersion while the gonioscopy findings did not reveal increased pigment and the angle was widely open in both eyes (Grade 4).

Phacodonesis is prevalent in highly myopic eyes.^10^ As literature suggests, insertion of a PC-pIOL further impairs zonular support as the lens floats, moves and rotates within the PC damaging zonular fibers especially during pupillary dilation.^10^

Finally, we faced a challenging cataract extraction case with the presence of phacodonesis as well as a rarely seen PC-pIOL. The explantation of the pIOL proved to be relatively easy with no need to cut or fold the lens. Excellent post-surgical VA was achieved by leaving the patient aphakic while avoiding implanting a zero diopter IOL since the patient was highly myopic. Phakic IOLs are important assets in refractive surgery as they help address the cases of high ametropia which cannot be dealt with otherwise. PC-pIOLs developed in 1986 with Fyodorov's pupil fixated design and it's evolution led to designs which have fewer side effects and improved outcomes.^1^^,^^2^^,^^5^ Without a doubt this prototype pIOL is rarely seen in practice today, however, fellow surgeons may be faced with a similar case. In this report, given the fact that literature is weak on this specific pIOL, we highlight the clinical manifestations associated with this pIOL as well as demonstrate our surgical technique showing the ease of its removal. Furthermore, being the first PC-pIOL ever used there is an additional historic value of this report.

## Patient consent

The patient consented to publication of the case in writing.

## Funding

No funding or grant support

## Authorship

All authors attest that they meet the current ICMJE criteria for Authorship.

## CRediT authorship contribution statement

**Spyridon Doumazos:** Writing – original draft, Data curation, Conceptualization. **Aikaterini Barlampa:** Writing – original draft, Data curation. **Stylianos A. Kandarakis:** Writing – review & editing, Formal analysis. **Menelaos Kanakis:** Resources. **George Kymionis:** Writing – review & editing, Validation. **Ilias Georgalas:** Supervision, Project administration. **Petros Petrou:** Writing – review & editing, Supervision, Conceptualization.

## Declaration of competing interest

The authors declare that they have no known competing financial interests or personal relationships that could have appeared to influence the work reported in this paper.

The authors have no conflict of interest.
